# If Plan A Does Not Work: The CD47 Ectodomain as a Target for Immune Tolerance

**DOI:** 10.3390/cells15010071

**Published:** 2025-12-31

**Authors:** Enrique Montero, Jeffrey S. Isenberg

**Affiliations:** 1 Department of Diabetes Immunology, 1500 Duarte Road, Duarte, CA 91010, USA; 2 Department of Diabetes Complications & Metabolism, 1500 Duarte Road, Duarte, CA 91010, USA; 3 Arthur Riggs Diabetes & Metabolism Research Institute, Beckman Research Institute, City of Hope National Medical Center, Duarte, CA 91010, USA

**Keywords:** CD47, TSP1, HLA-I, checkpoint, ectodomain, binding affinity, cancer, autoimmunity

## Abstract

Cell surface immune checkpoint receptors are objects for therapeutic intervention to stimulate immune cell attack of cancers. Interference between the checking ectodomain (ECD) and the natural ligand lowers constitutive restraints exerted on immune cells. This approach assumes that immune cells can do more, that a checkpoint blocker will make immune cells more effective at killing cancer cells, and that checkpoint molecules might have limited physiological roles. These assumptions may be warranted, as in the case of checkpoint-blockers towards the programmed death-ligand 1 (PD-L1) ECD, where clinical outcomes are consistently good. However, this does not appear to be the case for the universally expressed CD47 ECD. Much effort has been directed at engineering molecules that bind to the CD47 ECD to increase T cell and macrophage killing of cancers. But a wealth of clinical data do not indicate strong signals, improved killing, or meaningful survival advantages. This suggests that the CD47 ECD is a subpar target for cancer therapy. Consideration of reasons accounting for the modest benefits realized by molecules that bind to the CD47 ECD in cancer, also known as Plan A, is provided. This is followed by thoughts on what might be done, known as plan B, to identify advantages within the CD47 ECD for modulating tolerance in autoimmune diseases.

## 1. Introduction

Removing and disrupting the sense-of-self that standard surface antigens provide is one of the basic objectives of checkpoint therapies [1]. Checkpoint-directed molecules go after intrinsic immune inhibitory pathways that restrain T [2], NK [3,4], and dendritic [5] cells. This is especially true for CD47. Acting through SIRPα, the checkpoint action is embodied in macrophages as a ‘don’t-eat-me’ brake on phagocytosis [6]. The checkpoint paradigm found its best realization in the case of the PD-1/PD-L1, since the signal is narrow in its address, residing mostly within the cancer microenvironment [7]. For other checking pathways, ‘bleeding’ of the immune signal beyond the cancer is a significant issue [8,9] and a source of off-target complications [10]. The lack of local tumor effect and the tendency to generalize, besides accounting for the modest effects of some checkpoint blockers [11,12], imply that such genes are less critical for cancers to retain their self-status. The ability to leverage such pathways for cancer killing may be overly optimistic [13,14,15]. Data indicate that checkpoint molecules perform beyond suppressing immune cells. For instance, T-cell immunoglobulin domain and mucin domain containing molecule-3 (TIM-3) drives angiogenesis [16], lymphocyte activation gene 3 (LAG-3) is involved in lipid raft formation [17], T-cell immunoglobulin and ITIM domain (TIGIT) is adhesive [18], and PD-L1 participates in glycolysis [19]. Basic studies of these and other checkpoint genes have revealed insights into their roles in allo- and autoimmunity. For example, PD-1 antibody promoted alloimmunity and skin graft loss [20], and PD-1-null T cells exposed to alloantigen were more inflammatory [21]. The data gathered could serve as launch points for identifying means of quieting unwanted allo- and autoimmune activity. This may be the reverse side of the immune coin, but for the CD47 ECD, it could be the singular, practical side.

## 2. What Is CD47?

CD47 was identified in association with integrins using pull-down assays of tumor lysate [22]. The protein decorates all cells, and non-nucleated entities such as red blood cells and platelets [23], although the total copy numbers per cell vary by cell type [24,25]. While this results in differences in bulk expression, it is possible that, for any cell type, such as T cells, CD47 cell surface numbers, in other words the Bmax (maximum number of cell surface molecules), do not change much, or, if such changes do occur, they are short-lived [26]. This finding, if widely demonstrated in other cell types, would be consistent with the many jobs that CD47 has in arranging for cell homeostasis [27,28,29,30,31,32,33]. Moreover, 11 loss-of-function mutations are expected for CD47, but only one was observed [34], indicating a better than 90% chance that CD47 is loss-intolerant and maintains a key position in cell balance.

CD47 protein has multiple transmembrane domains that, at least in one location, link to the ECD [35]. This provides for a unique orientation between the ECD and the cell membrane. The ECD is multiply glycosylated [36], and the N-terminus shows a pyrrolidone carboxylic acid modification [37], which is needed for binding to one of its ligands. Heparan and chondroitin sulfate glycosaminoglycans also modify the ECD, and in T cells, this permits binding with soluble thrombospondin-1 (TSP1) [38]. Evolutionary conservation is suggested by the structural parity between the murine and the human CD47 ECDs [39] and by the findings that human TSP1 signals via rodent and higher mammal CD47 [31,40]. The diversity in the human CD47 ECD proteoforms from alternative splicing, single nucleotide polymorphisms, several iterations of its cytoplasmic tail, and post-translational adjustments likely manifest as a variety of CD47 ECDs on the same cell and between cell types [41], which could impact the interplay with soluble ligands [42] and other cell surface ECDs [26], as well as antibodies designed to bind to a particular ‘flavor’ of the ECD [43]. The CD47 ECD is also an object for sheddase cleavage [44], which could eliminate coupling with the ECD of another cell, although the signaling capacity of the cleaved CD47 ECD is unknown. Intriguingly, more CD47-SIRPα was noted in mono-cultures of non-immune cells under inflammatory stress [44]. But if this matters for the ‘don’t-eat-me’ signal between immune and non-immune cells should be tested in mixed cultures. Beyond SIRPα [45], the CD47 ECD interacts with other cell surface receptors such as vascular endothelial growth factor 2 (VEGFR2) [46], beta integrins [47,48], Fas receptor [30], CD14 [49], Rh blood group antigen complex [50], signaling lymphocytic activation molecule 7 [51], macrophage-1 antigen [52], and heterotrimeric G proteins [53]. In the latter, CD47 is linked to G protein-coupled receptors. It is currently thought that the CD47 ECD interacts in *cis* with the aforementioned ECDs. Parenthetically, the CD47 ECD seems to not be the only member of the checkpoint collection with *cis* activities. The SIRPα ECD also interacted in *cis* with beta 2 integrin [54]. However, the precise geometry, points of molecular contact, and structural realities of such binding events at the level of the crystal structure have only been described for the CD47 ECD in combination with the SIRPα ECD [55] and the CD47 ECD in combination with antibody B6H12 [35]. In addition, the CD47 ECD can interact in *trans* across the space between two cell membranes [56,57]. However, the significance of the *trans* coaction within a population of the same cell type versus a mixture of different cell types has not been weighed. That the *cis* versus *trans* fraternization of the CD47 ECD and other molecules has not been fully investigated, is perhaps because it is quite complicated, if not impossible, to control for all of the permutations. But data suggest that both *trans* and *cis* [58] signaling alter cells. Some of this was ascertained through cleavage of the *cis* SIRPα ECD, which increased inflammatory signals in a monoculture of human cells [58] and was supported by the finding that depletion of *cis* SIRPα on macrophages increased phagocytosis of liquid but not solid cancer cells [59]. Additionally, TSP1, the soluble high-affinity ligand of CD47 [31], efficiently blocked SIRPα binding to CD47 [31], a finding underappreciated but worth follow up. One might wonder if soluble TSP1 also upsets other CD47 ECD interactions. Alternatively, some regions of the TSP1 monomer may dimerize to promote post-translational modification and clustering of TSP1 [60]. This could drive aggregation of CD47 and *cis* and *trans* co-interactors. The crystal structure of TSP1 has not been solved. But the crystal structure of thrombospondin-2 (TSP2), a closely related family member that exhibits signaling similar to TSP1 [31], was reported [61]. The fact that TSP2 and TSP1 share signaling is expected, as their C-terminal domains, which bind CD47, are nearly identical [62]. In addition, the relative amounts and ratios of the other co-interacting molecules to the CD47 ECDs on any cell are not known. It is plausible that one or more than one of these is altered by agents, natural or engineered, that bind to the CD47 ECD.

Moving away from the ECD to the entire molecule, CD47 protein is translated from six gene transcripts [23,63], of which two yield active protein (see NM_198793.3). Differences in the 3′UTR of CD47 controls localization to the cell membrane versus the endoplasmic reticulum [64]. Several variations in the sub-membrane cytoplasmic part of the protein are known [39], and these may alter cell responses [65] or they be interchangeable without any distinct effect [66,67]. But the mapping and conditions under which each occurs, and how this modifies cell signaling, remain largely mysterious. Interestingly, alternate splice versions of the CD47 ECD have not been reported in humans. This would encourage the notion that the cytoplasmic domain of the molecule is a minor player in the business of the protein.

Whole CD47 protein appears to congregate on the cell membrane [68], although whether there is homophilic binding has not been thoroughly tested. Sometimes this is observed [69,70], while affiliation with other ECDs increases CD47 clustering [71]. Case in point, the SIRPα ECD dimerizes [72] and oligomerizes [73]. This also is well-characterized for other surface molecules [74,75]. Withal, the large amount of in vitro and rodent data that favor attacking the CD47 ECD ought to be weighed against the multitude of interrelations beyond SIRPα that the CD47 ECD is privy to.

## 3. Where Is CD47?

**Figure 1:**
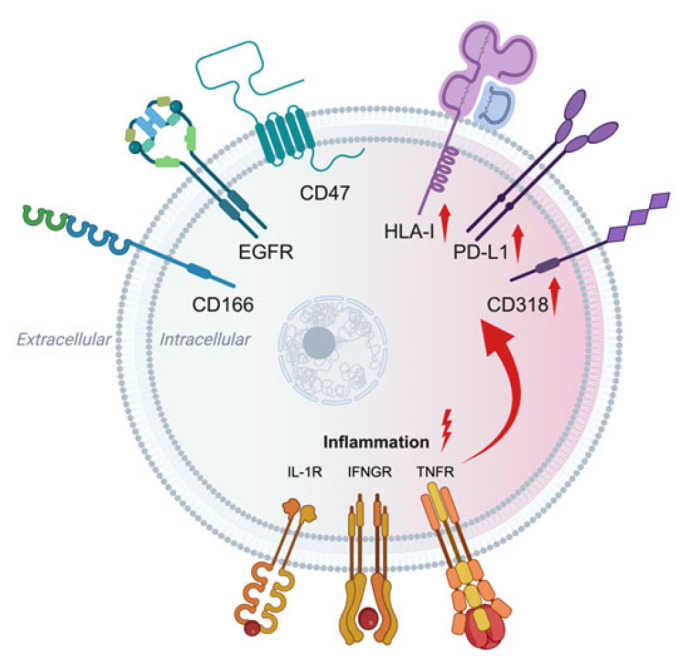
**CD47 and HLA-I are everywhere on human cells and are dynamically and differently modulated under pro-inflammatory stress.** The HLA-I ECD is present on all nucleated cells. Likewise, the CD47 ECD is found on every cell and on non-nucleated red blood cells [23] and platelets [94]. Human islet beta cells, the target of aberrant autoimmune damage in type 1 diabetes, showed diffuse expression of CD47 protein and transcript. Islet CD47 expression did not increase after short-term exposure to pro-inflammatory cytokines (IL-1β 50 U/mL, IFN-γ 1000 U/mL, TNF-α 1000 U/mL) [95]. Further, the cytokines did not increase CD166, which plays a role in cell activation and adhesion [96], and EGFR, a contributor to tissue homeostasis and proliferation, and anti-inflammatory. But, under similar pro-inflammatory conditions, HLA-I expression increased, along with other immunoregulatory surface proteins including PD-L1 and CD318 [97] and (Do JS, Chen W, Hung Y, Arribas-Layton D, Lu C, Gu A, Montero E, Carmo AM, Reijonen H. CD318 Expression Defines a Novel Subset of Human CD8^+^ Regulatory T Cells. Science Advances. 2025. Ms. No. adz4203. In press). Same cell *cis* SIRPα protein and transcript were not demonstrable on islet endocrine cells [95]. These data contrast with certain cancers where HLA-I goes down [98] and CD47 trends upward [23]. In the figure, the red shading denotes inflammation and the red-colored arrows denote increased protein expression mediated by pro-inflammatory cytokines. IL-1R, interleukin-1 receptor; IFNGR, interferon-gamma receptor, TNFR, tumor necrosis factor receptor.

CD47 [23] and human leukocyte antigen-1 (HLA-I) [76,77] are constitutively expressed on all mature nucleated human cells (Figure 1). Surface CD47 is found at different densities on several cell types [24]. And yet, a systematic analysis of cell surface and cytoplasmic CD47 molecules under inflammatory and metabolic stress, i.e., during pregnancy or infections, remains to be conducted. However, tracking the quantity of surface CD47 may be less important, as anti-CD3-activated human T cells did not show a change in the CD47 Bmax [26]. Similarly, restoration of surface CD47 in null T cells to control cell levels did not allow for changes in the Bmax following anti-CD3 activation [26]. The data imply that cells are strongly resistant to swings in membrane CD47. In addition, related to the ‘don’t-eat-me’ concept, the number of CD47 and SIRPα surface molecules varies between cell types and on the same cell [78]. That is, the CD47 and SIRPα ECDs are not found in a one-to-one ratio. On the basis of this and given that CD47 efficiently clusters SIRPα [78], there is reason to investigate the trend of saturating cells and organs with CD47 [79,80,81,82]. While this may be achievable in cell cultures and tissues from other species [83], the clinical realities of such an exercise as a means to improve transplant take remain elusive. Limited feedback indicates that CD47-plush porcine organ transplants fared well for several days under experimental settings in deceased recipients [80]. But they were not tolerated and failed in short order when transferred to living individuals [79,84]. If the CD47 ECD is more abundant in *trans* or *cis* than its immune cell ligand SIRPα, then why would more be better? Additionally, using excess CD47 ECD for transplantation does not square with results using CD47 blocking antibodies [85,86,87] and gene-suppressing molecules [88,89,90], all of which lower, as opposed to increase, effective CD47 signals. Under such circumstances, less CD47 ECD decreased transplant-related organ injury and improved survival. Interestingly, CD47 may contribute to alloantibody-mediated modulation of surface antigens and red blood cell (RBC) clearance following transfusion [91]. Non-nucleated RBCs do not express HLA-I [92], but they do express CD47, and the copy number changes with cell age [93].

CD47 appears to be dispensable for central T cell tolerance induction in the thymus and T cell repertoire formation [99]. However, CD47 contributes to maintaining peripheral tolerance by promoting T cell survival and function [100] and by regulating CD8^+^ T cell activation, proliferation, and fitness in a context-dependent manner [101]. That CD47 and HLA-I coexist on every nucleated cell is a circumstance whose meaning may not have been considered in peripheral immune regulation. HLA-I presents immune cells molecules to protect against foreign invaders and to support tumor immune surveillance [102,103]. Could one of the molecules presented by HLA be a peptide from relevant CD47 ECD binding locations? If so, would this be a means of supplementing cell surface CD47 signals for homeostasis? The implication here would be that HLA-I acts to maintain immune balance by showing T cells, which carry cell surface ligand SIRPα [104,105], the recognition signal contained in the CD47 ECD. This idea is not unreasonable, given that CD47 ECD-derived peptides, 8 to 21 amino acids long, showed signaling activity [106,107]. One issue is whether the HLA-I ECD binds and displays a CD47 ECD peptide fragment. A second question is whether such CD47 ECD fragments are processed within the cell. All of this is rendered more defensible by the observation that HLA peptide cross-presentation occurs [108], and also supported tangentially by the occurrence of CD47 autoantibodies [109]. Plus, individuals with systemic lupus erythematosus had circulating natural CD47 autoantibodies, while the level of immune cell CD47 correlated with the severity of inflammation [110]. Altogether, one theorizes that autoimmunity to CD47 as a self-antigen contributes to peripheral immunoregulation and tissue homeostasis, making it a candidate for the immunological homunculus [111,112,113].

## 4. How Does the CD47 ECD Work?

Consequent to alternative splicing, there are several versions of the cytoplasmic tail of CD47 that vary in expression by cell type [63]. However, transactions between any of these and cytoplasmic molecules are few. Using yeast hybrid assays, partnering between the cytoplasmic end of CD47 and Bcl-2 homology 3 (BH3)-only protein 19 kDa interacting protein-3 (BNIP3) was noted [114], suggesting that CD47 advances apoptosis and cell death. Interestingly, TSP1, but not SIRPα, signaled via CD47-BNIP3 [114]. The CD47-BNIP3 signal required the cell membrane region of BNIP3, providing another example of lateral *cis* communication via CD47 [114]. The other direct connection between CD47 and the cytoplasm is via ubiquilin-1 and ubiquilin-2 [115], which links CD47 to the cytoskeleton. Still, the minimal known direct connections between CD47 and submembrane pathways are consistent with its capacity to organize other receptors. The finding that the CD47 ECD alone and in *cis* recruited and activated integrin alpha v beta 3 [48,116] bolstered the idea that CD47 works mainly via *cis* and *trans* interplay with other ECDs. Similarly, the soluble CD47 ECD was sufficient to activate integrins, indicating *trans*-mediated agency [116]. Thus, the CD47 ECD binds to the ECDs of other surface molecules and promotes their signaling. It is worth pointing out again that the CD47 ECD has dealings with several receptor families including the tyrosine kinase family (i.e., VEGFR, SIRPα), the tumor necrosis factor receptor family (Fas receptor), the leucine-rich repeat protein family (CD14), G protein-coupled receptors (via heterotrimeric G proteins), the signaling lymphocytic activation molecule family (via member 7) [51], and in red blood cells, via the Rh blood group antigen complex, a link to the cell cytoskeleton. This latter dynamic has a hand in age-related red cell changes in deformability associated with cell clearance and thus can be considered part of the ‘don’t-eat-me’ signal [117]. On a related note, the CD47 ECD participates with soluble ligand to alter Ca^2+^ transfer into the cytoplasm of red blood cells [117] and other cell types [40,118]. The binding affinities between the CD47 ECD and other ECDs are well determined in only a few instances and are summarized in Table 1.

## 5. Puzzles Regarding CD47 ECD Antibodies and CD47-SIRPα

This brings up puzzles arising from exploiting the CD47 ECD with antibodies, the dominant clinical strategy (Table 2). The first conundrum is apparent, namely that research- and clinical-grade CD47 antibodies were not developed with regard to, and do not discriminate among, the soluble and other ECD ligands that engage the CD47 ECD. Thus, the mechanisms attributed to these antibodies will remain indecipherable since the effects on other ECDs and cytoplasmic signaling cannot be accounted for. A derivative of this is the idea that an antibody binding to the CD47 ECD undoes the constitutive brake on immune cells attributed to SIRPα. The logic and data behind this are problematic. For example, SHP1/SHP2, the canonical downstream effectors of SIRPα activation, are also targets of indispensable genes, such as mitogen-activated protein kinase 1 (MAPK) [123], which captures ERK [124], JNK [125], FcγRIIa clustering [126], and others [127]. These genes are themselves linked to immune cell activation [128,129,130], but controlled studies have yet to identify SIRPα-specific changes. Also, in some cases, a lack of SIRPα or expression of a SIRPα mutant, that did not activate SHP1/SHP2, was actually anti-inflammatory [131].

A second area of confusion is that macrophage uptake can actually be triggered and increased by cell surface CD47. In other words, phagocytosis was enhanced if the target cells displayed the CD47 ECD [135]. This was true even when the CD47 ECD was clustered on the surfaces of the target cells [135], which, in theory, should enforce the SIRPα phagocytosis-limiting signal. The pro-phagocytotic activity of CD47 was found in the absence of the “eat-me” signal of phosphatidyl serine [135]. Incidentally, the target cells were apoptotic and CD47 ECD clustering was lower in apoptotic cells [136]. Again, removing the CD47 ECD on murine lymphoma cells rendered them protected from phagocytosis, while replacing the CD47 ECD on the cells led to their engulfment [146]. In a further twist, the lack of or retention of CD47 EDC made absolutely no difference in phagocytosis, a result revealed by restricting serum in the culture media [147]. This conjures up serum factors as a way to ascribe a role for the CD47 ECD as a checkpoint. It was postulated that the target cell CD47 ECD, via macrophage SIRPα, served as a lanyard to keep the cells in place to permit phagocytosis [147]. As an aside, it was found that the soluble CD47 ECD phosphorylated SIRPα [147]. In a similar manner, SIRPα brought about phagocytosis [148]. Obfuscating understanding is the finding that macrophages lacking the SIRPα ECD, but that had the CD47 ECD, were not more phagocytic [51], which casts the *cis* mechanism of immune regulation in a suspect light. One can imagine how this impinges on cell and animal studies employing engineered CD47 ECD-binding molecules. The premise has been that CD47- and SIRPα-ECD-binding antibodies disrupt a brake on macrophages and other immune cells. In light of contradictory findings that CD47 and SIRPα can promote phagocytosis, an alternate hypothesis is that CD47 and SIRPα ECD-binding antibodies instigate a primary signal that promotes macrophage activation and phagocytosis. The suppression of autoimmune sarcoidosis by an antibody that blocks CD47-SIRPα supports this concept [149].

Still unsolved is the presumed primacy of CD47 in phagocytosis. The CD47 ECD is not sufficient, as clinical antibody data amply highlighted, and is not needed for phagocytosis, as some cell studies indicated. This is secondary to the many side roles that CD47 plays, such as with beta-1 integrins. However, the latter themselves direct phagocytosis [150,151,152], and this side of CD47 has not been controlled for in any studies of phagocytosis. The CD47 antibody B6H12, a precursor for the clinical CD47 antibody Magrolimab, activates beta 1 integrins [132]. Increased phagocytosis revealed upon treating with this antibody could represent a beta-1 integrin effect and be entirely unrelated to SIRPα. While on the topic, integrins are required for SIRPα activation [153]. Therefore, a CD47 antibody that alters CD47-SIRPα binding may act directly via integrins to arouse phagocytosis, and indirectly via integrin activation of SIRPα.

CD47 antibodies differentially impact the systemic immune response [154]. Rather baffling is why certain CD47 antibodies result in less RBC phagocytosis than others. A bivalent CD47 antibody was found to clump red blood cells [155], which would be expected to also cluster CD47 ECDs. Restructuring the molecule to be monovalent resulted in less anemia, presumably without a decrease in phagocytosis of non-red blood cells [155]. Another example is a bivalent SIRPα antibody that binds the CD47 ECD [156]. It did not bind more than a nonspecific control to cells with CD47. Since the affinities seemed to be the same, the question arises as to how much the natural state of things was disrupted. But behind all of these efforts is the unstated assumption that the two distinct cell types can have CD47 ECDs that vary in structure to favor selective binding to the target. This sort of thing is found in immune cell receptors via recombination [108,157] but has not been reported for the CD47 ECD. Adding to the Gordian knot of the CD47 ECD are data showing that *cis* CD47-SIRPα limits phagocytosis as well, and perhaps better, than the *trans* signal [158]. Then, which is it, and why the redundancy?

Human TSP1 bound to human T cell CD47 with an estimated Kd of 12 picoM [31]. This remains the highest binding demonstrated for TSP1. In so doing, TSP1 blocked SIRPα binding to CD47 [31]. As a 450 kD trimer, TSP1, at modest concentration, could blanket CD47 to exclude SIRPα or disengage any existing CD47 attached to the SIRPα ECD. TSP1 is increased in hypoxia [159], inflammation [160], and cancer [133,134], times and places where immune cells are active and embody different immunoregulatory tendencies. TSP1 provoked macrophage killing of cancer cells [161,162]. More work is needed to distinguish between TSP1-mediated disruption of the CD47 ECD with SIRPα and its contribution to promoting phagocytosis, versus other less-specific pro-inflammatory signals, such as superoxide that TSP1 stimulates [163], which is pro-phagocytotic [164,165]. Or TSP1 could take control of SIRPα. In primary human cells, TSP1 assumed a proactive stance and phosphorylated SIRPα and SHP1/2 [119]. This would be consistent with TSP1 having an overall phagocytic tendency. There is also, waiting on the sidelines, surfactant protein D, an immune cell regulator [166], which binds the ECD of SIRPα [167], but whether this impacts immune cell activation via CD47, while put forward, was not tested [168].

Perplexing is why normal quantities of the CD47 ECD are enough to enforce a brake on phagocytosis, yet to gain immune approval of cells and organs from other sources, extraordinary amounts of the ECD are deemed essential and applied [137,138]. A match in binding affinity between the CD47 ECD and the target ECD was all that was needed to arrive at tolerance [139]. Then, the deployment of supraphysiologic CD47 ECD should be superfluous. The apparent binding increased with the number of CD47-SIRPα complexes, suggesting a cooperation [78], which may help when excess CD47 ECD is employed. Modeling pictured that the cooperative phase of the CD47/SIRPα complex occurred with out-of-plane membrane fluctuations. As a result, low expression levels of CD47 or SIRPα alleles with less affinity would achieve sufficient levels of self-signaling to limit phagocytosis [78]. As an aside, the superabundance of CD47 forced on allogeneic cells is never found in a vacuum [140] but is always in conjunction with altered expression of other surface antigens (B2M null, CIITA null) [141] and with xenogeneic organs (GGTA1, Β4GalNT2, CMAH, CD46, CD55, TBM, EPCR, HO-1) [169]. However, factorial testing of the ECD combinations should be carried out. Opposing these data, in models of excessive CD47, transplanted CD47-null hearts survived better in situations of MHC mismatch [142], while CD47 ECD blocking antibodies mitigated whole-organ ischemia–reperfusion injury [143] and enhanced transplant survival [144]. This approach was also useful in porcine organ donation-after-death transplantation [145] and in preserving organ health ex vivo under machine perfusion [170], suggesting a niche in organ procurement and transplant bridging.

## 6. How Is CD47 Being Intersected (Also Known as Plan A)?

The clinical agents targeting the CD47 ECD were inspired and based upon published data in cells and mice that employed the research-grade CD47 antibody B6H12 [122,171,172]. An early finding was that a fragment of B6H12 blocked neutrophil migration [173]. Interestingly, B6H12 cross-linked the CD47 ECD, increased T cell proliferation and CD25 expression, and phosphorylated the p56(lck) protein tyrosine kinase [174], all of which are implicated in increased T cell activity. B6H12 stimulated several beta 1 integrin adhesion pathways in T cells [132]. In other cells, B6H12 drove cell cycle arrest and inhibited proliferation [175]. Separate from checkpoint actions, B6H12 blocked cancer cell proliferation [176], pro-growth VEGF [122], and nitric oxide signaling [177]. Variations on this theme continue to be explored [178,179]. They all share a common lineage and seek to step into the CD47 ECD-SIRPα ECD partnership, separate from the many other ECDs with which CD47 interacts. But these facts give satisfactory reasons to avoid the ECD. The closure of CD47 ECD antibody programs by large pharmaceutical companies (see NCT05626322 and others) could be anticipated given contradictory data showing that while the loss of CD47 ECD led to SIRPα activation, this was insufficient to account for the increased tumor clearance observed in rodent studies [180,181], and assumes more value in the models used over the evolutionarily conserved functions of the CD47 ECD with protein sequence homology across species [39,182,183]. Still, optimism persists highlighted in over a thousand scientific papers and several hundred summaries found on the PubMed search engine that cover antibodies and other molecules to the CD47 and SIRPα ECDs. But the clinic has not yielded worthy outcomes for people with cancer. Binders of the CD47 ECD are also in play in outside of cancer, such as in atherosclerosis [184], an idea previously validated to be feasible [90,185]. Whether use of CD47 ECD antibodies can remodel the walls of damaged arteries and restore capillary networks in the atherosclerotic vasculature of people is an interesting question. The outcome could be countered by the undesirable effects of CD47 ECD binders on autoimmunity, which are shared with other checkpoint-blocking molecules [186], and by agnostic signaling via CD47 in non-cancer cells that limits essential processes [187,188]. It is also the case that long-term use of these agents may open a window for cancer [189]. Attention is turning to the view that the CD47 ECD is not simply a convenient means of keeping SIRPα happy [190]. The interplay with other ECDs and with TSP1 [191] may never be divorced from CD47 [56,192]. The Plan A way of reaching out to the CD47 ECD would appear not suitable.

## 7. How Might CD47 Be Improved Upon (Also Known as Plan B)?

Enthusiasm in fashioning CD47 therapeutics likely stemmed from the successful experience with targeting other surface ECDs with antibodies [193,194,195,196,197,198]. It would be expected that such results might transfer to the development of molecules against other immune-linked ECDs. But as enumerated above, CD47 is not merely another immune-linked cell surface ECD. It is a circuit box that works to keep the cell in a balanced state. High-affinity CD47 antibodies are preferentially selected for immunotherapy [199] as in other targeted models, including EGFR [200]. However, lower-affinity molecules that emulate natural autoantibodies may match their therapeutic effect with reduced side effects [200], suggesting the importance of avidity and fine specificity.

To avoid the legion of additional CD47 ECD connections, co-associations, and binding events, two alternatives are offered. The first, Plan B1, would involve suppression of CD47 protein production. Secondary to the degradation of existing CD47 protein, this approach would gradually lower total CD47. This is attainable via several means, such as with molecules that block translation of mRNA [201] or that promote exon skipping [202]. These types of molecules are in the clinic, albeit for other purposes. Whether Plan B1 would favor more or less immune cell activity may depend upon the context. The second, Plan B2, would flip the ECD on its head, so to speak, and use it not to upset immune cells, but as a way to quell the cells when overactive immunity is the disease. An abundance of soluble CD47 ECD will be anti-inflammatory, but for reasons less suspected [56]. This position was implied in the finding of more and accelerated autoimmune diabetes in mice with aberrant CD47 ECD binding dynamics [203]. The target is not the CD47 ECD, since, as emphasized before, there are no indications of CD47 ECD homodimerization. The targets would be ECDs that combine with the CD47 ECD. Furthermore, soluble CD47 ECD could stick to TSP1 neutralizing its ability to act in an inflammatory manner with surface CD47.

Plan B1 and Plan B2 would side-step the possibilities that (i) cell surface CD47 coverage cannot be controlled with soluble ECD antibodies; (ii) that too much interference with CD47 ECD binding decreases phagocytosis [204]; and that (iii) CD47 ECD ligands will behave as TSP1 agonists, upsetting cell equipoise [205]. Plan B1 and Plan B2 could revive the CD47 ECD as a therapeutic.

## 8. Summing Up Our Point of View

What we are seeing in the case of CD47 is a surface protein that has minimal direct links to events below the membrane but instead sets the stage for other cell receptor signals to operate. CD47, via the co-interactions with the ECDs of other receptors, provides a means for optimal signaling. But with so many relationships, it is unclear whether artificial molecules that bind to the CD47 ECD will ever be safe or effective. Lessons from CD47-specific autoantibodies with potential immunoregulatory roles should be considered. This asks us to exercise caution and re-evaluate the rationale for using antibodies as a key to CD47-based cancer therapy. Beyond that, soluble CD47 ECD, alone and separate from the whole protein, should not disrupt the many established states among CD47 and its associated receptors. Indeed, the CD47 ECD does not show homophilic binding. It may hold potential on the other side of the coin: the CD47 ectodomain as a target for immune tolerance in alloreactivity and autoimmune diseases, including pancreatic islet transplantation and type 1 diabetes.

## 9. Departing Questions

What is the purpose of the SIRPα ECD in a world where the CD47 ECD is in demand by many other ligands and at higher affinities (Table 1)?

Do CD47 natural autoantibodies share similar specificity with existing high-affinity engineered molecules? Are the former polyreactive modulating the interaction of CD47 with a wide range of its ligands?

Although CD47 and HLA-I are almost always found together on all human nucleated cells, only HLA-I increases under pro-inflammatory stress, facilitating antigen presentation (Figure 1, Graphical Abstract). Does CD47 overexpression pose a risk to reduce the protective immune response in nature?

## Figures and Tables

**Table 1:** **CD47 ECD binding affinities.**

Interaction	Affinity	Method	Cells/Protein	Reference
Natural				
TSP1-CD47 *	Kd 12 pM	radiolabel competition	^125^I-hSIRPα, hTSP1, hCD47, T cells	[31]
TSP1-SIRPα ^(a)^	NA	radiolabel competition	Radiolabeled ^125^I-hSIRPα in buffer; hTSP1 on plate	[119]
CD47-SIRPα	Kd 1 µM (1)	SPR	hCD47, hSIRPα	[120]
	Kd 1.2 µM (2)	SPR	hCD47, hSIRPα	[36]
	Kd 0.2 µM (3)	SPR	hCD47-CD4-6His, hSIRPα-biotin	[121]
CD47-SIRPα	Kd 0.08 µM	SPR	hCD47, mSIRPα	[120]
CD47-SIRPα	Kd > 30 µM	SPR	mCD47, hSIRPα	[120]
CD47-SIRPα	Kd 4.7 µM	SPR	mCD47, mSIRPα	[120]
CD47-VEGFR2	NA	FRET	CD47-GFP, VEGFR2-mCherry, and CD47 + VEGFR2 were transfected into HUVEC, hTSP1	[46]
TSP1-VEGF	NA	hTSP1 on plate	hTSP1, ^125^I-VEGF	[46]
CD47-β integrin	NA	Pull down assay	hαIIbβ3, hCD47	[101]
**Engineered**				
CD47-SIRPα	Kd 0.01 µM	colorimetric	hCD47 ECD fused to alkaline phosphatase, hSIRPα expressed in CHO cells	[73]
TSP1 signature domain-CD47	competition assay		^125^I-E123CaG1, T cells	[31]
CD47-CD47	no binding	SPR	bivalent hCD47-Fc	Unpublished, the authors
CD47-Magrolimab ^(b)^	Kd 1 8–19 nM monovalent Kd 2–14 pM bivalent	SPR	hCD47-murine Fc	[122]
CD47-B6H12 ^(c)^	NA	NA	engineered human CD47 hybrid with Escherichia coli (BRIL) inserted into loop 1 of the intracellular loop	[35]

hCD47, hSIRPα, human protein; mCD47, mSIRPα, murine protein. * CD47 implicated in interactions with SLAM7, Fas, CD14, Rh complex, and MAC1, but binding affinity (Kd) data is not available. Caveats: ^(a)^ readout: y axis cpm radioactivity; x-axis TSP1 concentration; ^(b)^ not human Fc; ^(c)^ only crystal structure interactions reported.

**Table 2:** **Some CD47 ambiguities.**

	Ambiguity	Observations and Comments	References
1	CD47 Abs do not discriminate between the CD47 ECD and interaction with other ECDs.	A CD47 Ab may disrupt or prevent natural interactions between the CD47 ECD and other ECDs.	[31,46,47,48,49,50,52,53]
2	CD47 Abs activate integrins.	Integrins drive phagocytosis. Thus, the role of CD47-SIRPα could be a moot point.	[116,132]
3	CD47 Abs do not bind CD47 ECDs equally.	It is not clear if this is because the CD47 ECDs on one cell type vary structurally versus those on another cell type, or because ECDs are the same and the Abs vary in affinity.	[133,134]
4	SIRPα is a minor activator of SHPS1/2.	The effectors of SIRPα are targets of core cell circuits run by ERK and MAPK. The data on the role of SIRPα related to CD47 may be related to pathways driven by ERK and MAPK.	[123,124,125]
5	CD47 is not needed for protection from phagocytosis.	Cancer cells with and without CD47 are phagocytized. The CD47 ECD is not sufficient or necessary to suppress macrophages.	[131,135,136]
6	SIRPα is not needed for phagocytosis.	SIRPα-null macrophages phagocytosed equally well compared to SIRPα cells.	[51]
7	Excess CD47 is added artificially but does not protect.	CD47 is a natural break on many immune cells; why is more needed in transplants? CD47 ECD binds one-to-one with immune cell SIRPα ECD, so what does extra CD47 interact with?	[137,138,139,140,141]
8	CD47 loss and blockers improve transplantation.	Organs that are CD47-null survive and flourish after transplantation. CD47 blocking Abs improve transplants.	[142,143,144,145]

## Data Availability

In the present study, new data was not created or analyzed. Data sharing is not applicable.

## Artificial Intelligence

AI was not employed in the creation of any part of the manuscript.
